# Cholesin, a new hormone bridges intestinal cholesterol absorption and hepatic synthesis

**DOI:** 10.1093/lifemeta/loae024

**Published:** 2024-06-07

**Authors:** Peter U Amadi, Da-Wei Zhang

**Affiliations:** Department of Pediatrics, Group on the Molecular and Cell Biology of Lipids, University of Alberta, Edmonton, AB T6G 2S2, Canada; Department of Pediatrics, Group on the Molecular and Cell Biology of Lipids, University of Alberta, Edmonton, AB T6G 2S2, Canada

**The balance between intestinal cholesterol absorption and cholesterol synthesis in the liver has remained a puzzling phenomenon for a long time because no study has been able to explain how the consumption of a cholesterol-rich diet impairs cholesterol synthesis in the liver. Recently, a group of researchers uncovered a hormone produced in the intestine without any known function, which they named cholesin, that suppresses sterol regulatory element-binding protein 2 (SREBP-2), the master regulator of cholesterol synthesis in the liver.**

Several tissues work in synergy to manage the balance between the absorption, synthesis, and excretion of cholesterol. This balance is critical because high circulating cholesterol levels, especially the low-density lipoprotein cholesterol (LDL-C), can cause severe cardiovascular consequences [1]. The cholesterol carried in LDL is mainly provided by *de novo* cholesterol synthesis and absorption from the gut. Cholesterol absorption most accurately refers to the transfer of intraluminal cholesterol into the intestinal lymph. The small intestine plays a central role in providing cholesterol to the body through several sources. Cholesterol enters the intestinal lumen from the diet, epithelial slough, or bile. The bile contributes the greatest amount of absorbed cholesterol of up to 1200 mg/day, while the diet and epithelial slough contribute about 500 and 300 mg/day, respectively [2]. Approximately 400 mg/day of endogenous and exogenous cholesterol are metabolized into bile acids and 50 mg/day into steroid hormones while the rest is excreted through the skin and feces [3]. Because cholesterol is almost insoluble in aqueous environments, it forms complexes with bile salt solutions in the intestinal lumen to diffuse through the intestinal lumen-membrane barrier before uptake by cholesterol transporters at the brush border of enterocytes.

One of the most studied cholesterol transporters at the enterocyte is the Niemann-Pick C1-like 1 protein (NPC1L1) expressed at the apical surface of enterocytes [4, 5]. Only non-esterified cholesterol can be trafficked into the enterocytes by cholesterol transporters and secreted into the intestinal lymph as cholesteryl esters, implying that the cholesterol-esterifying capacity of the intestinal enterocytes regulates cholesterol absorption in the gut. Inside the enterocytes, the majority of cholesterol becomes esterified at the endoplasmic reticulum (ER) by acyl-coenzyme A (CoA):cholesterol acyltransferases (ACATs) and along with phospholipids and triglycerides, is added to apolipoprotein B48 to form nascent chylomicron particles. Before exocytosis of chylomicrons from the enterocytes, they are transported from the ER to the Golgi apparatus, where they are transformed into mature chylomicrons [6]. In circulation, lipids, mainly triglycerides on chylomicrons, are hydrolyzed by lipoprotein lipase and are provided to tissues such as the cardiac, adipose, and skeletal muscle tissues. The extensive hydrolysis of the triglyceride core forms chylomicron remnants, which, alongside the constituent cholesterol, are taken up by the liver.

The liver contributes to the majority of *de novo* cholesterol synthesis in the body. Cholesterol synthesis initiates in the ER from acetyl-CoA, through multiple steps that are highly regulated at the transcriptional and posttranslational level. The most important step in *de novo* cholesterol synthesis is the conversion of 3-hydroxy-3-methyl-glutaryl-CoA (HMG-CoA) to mevalonate catalyzed by HMG-CoA reductase (HMGCR). The expression of HMGCR and several enzymes involved in *de novo* cholesterol synthesis is primarily regulated by a protein called the sterol regulatory element-binding protein 2 (SREBP2) at the transcriptional level [7]. This regulatory process is highly dependent on cellular cholesterol levels. When cellular cholesterol levels are low, SREBP2 is activated stepwise: transferred from the ER to the Golgi apparatus, where it undergoes a sequential cleavage by site-1 proteinase and site-2 proteinase to release its transcriptional active form. This exposes a nuclear localization signal in SREBP2, allowing its transcriptional active form to travel to the nucleus, where it serves as a transcription factor that enhances the transcription of *HMGCR* and other genes involved in cholesterol metabolism. The activation of SREBP2 also enhances the transcription of LDL receptor (*LDLR*) and proprotein convertase subtilisin kexin 9, both of which are directly involved in the regulation of cholesterol homeostasis. On the other hand, when cellular cholesterol exceeds normal levels, cholesterol induces the formation of the SREBP cleavage-activating protein (SCAP)/insulin-inducible gene protein (INSIG) complex, causing the retention of SREBP2 in the ER and inhibition of the transcriptional activity of *SREBP2* [8]. Another important factor that regulates circulating cholesterol levels is the crosstalk between key regulators of intestinal cholesterol absorption and *de novo* cholesterol synthesis in the liver. This is evident from studies showing that cholesterol synthesis is affected by dietary intake of cholesterol-rich diets and cholesterol interventions. Notwithstanding this, the key factors that govern this coordinated response of the liver’s cholesterol synthesis to intestinal cholesterol absorption have not been defined in literature until Hu and his group identified the hormone responsible for this, which they named cholesin.

Until now, there has been no known function of cholesin secreted from the intestine during the intestinal absorption of cholesterol [9]. The group showed that after 4 h of high cholesterol administration, the expression of HMGCR in the intestine was downregulated. This downregulation resulted from negative feedback of cholesterol, inhibiting its own synthesis. In addition to this, the transcription and protein levels of hepatic HMGCR significantly and rapidly decreased, suggesting that additional factors regulated hepatic cholesterol synthesis other than intestinal cholesterol. To substantiate this, the group conducted a fasting-refeeding study and analyzed plasma proteins from mice that fasted for 16 h and refed for 1 h with a regular or Western diet. Silver staining analysis revealed a 23 kDa protein band in the group of mice refed with the Western diet. This protein was later identified as an uncharacterized highly conserved human protein, the C7orf50, which they named cholesin. Additionally, the group demonstrated that cholesin was secreted in the plasma of refed mice and humans and was highly expressed in the intestine and colon. To further demonstrate that the production of cholesin was indeed in response to cholesterol intake, the group generated transgenic mice lacking cholesin in the intestine and performed the 1 h refeeding study with a Western diet following a 16 h fasting, or an oral gavage of cholesterol. They observed, with these transgenic mice, that neither the Western diet nor cholesterol oral gavage stimulated as much plasma cholesin levels as normal mice, and despite this, the cholesterol content in the intestines remained comparable between the normal and transgenic mice. Using human colorectal carcinoma cells that stably expressed cholesin, the group also observed that cholesin was secreted to the medium proportional to varying doses of administered cholesterol, and this process was impaired with *NPC1L1* knockdown, the intestinal cholesterol transporter. This justified their hypothesis that cholesin secretion is reliant on NPC1L1-mediated uptake of cholesterol.

Interestingly, the authors found that plasma cholesin levels in humans were negatively correlated with plasma total cholesterol, triglycerides, apolipoprotein B (APOB), and LDL-C levels, but found no relationship with high-density lipoprotein cholesterol (HDL-C) or its apolipoprotein, APOA1. To further buttress these findings on the relationship between cholesin and plasma cholesterol and triglycerides, the authors observed that the transgenic mice lacking intestinal cholesin produced significantly elevated plasma and hepatic cholesterol and triglycerides following a Western diet feeding. However, using fast protein liquid chromatography to examine plasma lipoproteins from the transgenic mice, the authors observed that HDL-C was also elevated alongside LDL-C and very low-density lipoprotein cholesterol (VLDL-C). Because the increase in plasma cholesterol resulting from intestinal cholesin deficiency may have resulted from several factors such as increased absorption from the intestines, hepatic synthesis and VLDL secretion, or impaired cholesterol clearance and biliary excretion, the authors investigated the major mechanisms behind the cholesin-mediated elevation in plasma cholesterol. Increased intestinal absorption was unlikely due to similar intestinal cholesterol and triglyceride levels between the transgenic mice lacking cholesin and normal mice, as well as no significant differences in food intake and fat mass. Also, the rate of chylomicron catabolism, fecal contents of cholesterol and triglycerides, and bile acid and biliary cholesterol levels were all unchanged in the transgenic mice compared to normal mice, excluding cholesterol clearance and biliary excretion as the likely mechanism behind the increased plasma cholesterol in the transgenic mice. The increase in the transcription of *SREBP2* and other genes that mediate cholesterol synthesis indicated that the elevated plasma cholesterol resulted from increased hepatic cholesterol synthesis. They further injected the transgenic mice with poloxamer-407, an inhibitor for lipoprotein lipase, and after feeding these mice with a Western diet, they observed elevated plasma triglycerides and APOB, which implied that cholesin deficiency also increases triglycerides by enhancing hepatic VLDL secretion.

The next question the authors needed to answer was, like every other hormone that functions through a receptor, how does cholesin mediate its effect on hepatic cholesterol? Firstly, the authors attempted to identify the target tissue for cholesin by incubating several mouse tissues with purified GST-tagged cholesin and observed that the liver, kidney, adipose, and skeletal muscle tissues were the most likely cholesin target tissues. Next, the authors compared the results of flow cytometry of different cell lines that bind cholesin and cells with cholesin binding deficiency and found that cholesin may mediate its effect through a conserved G-coupled protein receptor called GPR146, which has been reported to regulate circulating cholesterol levels [10]. To elucidate this further, the authors demonstrated that tissues that originally had higher cholesin binding affinity lost this capacity when isolated from transgenic mice lacking *Gpr146*. Furthermore, the authors replaced six amino acids to generate a mutant form of GPR146 (Mut GPR146) that produced a lower cholesin binding affinity and was unable to restore the affinity for cholesin in hepatocytes isolated from *Gpr146* deficient mice. To investigate whether cholesterol synthesis was inhibited by cholesin in a GPR146-dependent manner, the authors injected cholesin dose-dependently and observed a decrease in hepatic and plasma cholesterol, as well as the expression of genes that regulate cholesterol synthesis. This effect was blunted in transgenic mice lacking *Gpr146* expression in their hepatocytes (*Gpr146* LKO) but became restored when hepatic expression of *Gpr146* was upregulated with the Adeno-associated virus. Because earlier studies showed that GPR146 signaling is involved in the extracellular regulated kinase 1/2 (ERK1/2) pathway given that *Gpr146* deficiency in the liver diminishes ERK1/2 activities, the authors attempted to investigate whether the cholesin effect on cholesterol was mediated through the ERK1/2 pathway. Cholesin administration significantly decreased ERK1/2 phosphorylation (pERK1/2), which was restored with the addition of wild type but not the mutant GPR146.

**Figure 1:**
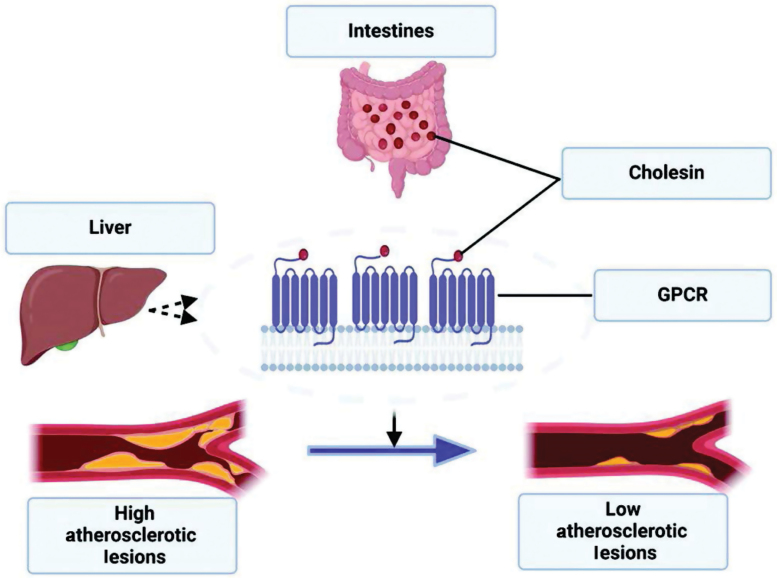
Schematic diagram of cholesin-mediated regulation of hepatic cholesterol metabolism leading to improved atherosclerotic lesions. Cholesin is secreted by intestinal enterocytes, binds to G-protein coupled receptors (GPCRs) of the liver cells, and elicits a signaling process that decreases hepatic cholesterol synthesis and plasma cholesterol levels thereby ultimately improving atherosclerosis and hypercholesterolemia.

Finally, despite the breakthrough recorded with the use of statins to manage hypercholesterolemia, one major concern yet to be addressed was the statin-mediated increase in cholesterogenic genes. Thus, therapies that provide cholesterol-lowering effects through mechanisms that exclude the elevation of cholesterogenic genes represent promising candidates. Considering that cholesin administration suppressed the expression of cholesterogenic genes, the authors investigated the atheroprotective potential of cholesin during hypercholesterolemia and atherosclerosis. Cholesin was administered exclusively or in combination with a statin called rovustatin, to transgenic mice lacking LDLR, a well-established model of atherosclerosis. The administration of cholesin significantly lowered plasma cholesterol and expression of hepatic cholesterogenic genes in dyslipidemic mice. The authors showed that similar to cholesin, rosuvastatin administration decreased plasma cholesterol but upregulated the expression of cholesterogenic genes. When cholesin was administered together with rovustatin, the authors observed that the rovustatin-induced increase in the expression of cholesterogenic genes was abolished, thereby allowing a more robust inhibitory effect on plasma cholesterol and atherosclerotic lesions (Fig. 1). In addition, a combination of statin and cholesin decreased body weight gain, lipid accumulation, and inflammation in the liver, and lowered plasma triglyceride levels.

In summary, Hu *et al*. provide compelling evidence supporting the notion that cholesin-GPR146 mediates the inhibitory effect of intestinal cholesterol absorption on hepatic cholesterol synthesis. These new findings expand and deepen our understanding of the impact of communication between different tissues and cells on overall cholesterol metabolism. Furthermore, these findings may have provided a strong rationale behind the potential therapeutic effects of cholesin for the management of hypercholesterolemia and atherosclerosis. To achieve this goal, more research is warranted to gain a clearer understanding of the underlying molecular mechanisms. For example, (i) Hu *et al*. reported that in addition to the small intestine, cholesin is also highly expressed in the stomach and colon of mice. The protein is also expressed in the mouse brain. Human Protein Atlas data show that in addition to the gastrointestinal tract, C7orf50/cholesin is also expressed in various tissues in the human body, such as skin, kidneys, and tonsils. What is the role of cholesin in these tissues? (ii) The authors reported that GPR146 is a functional receptor for cholesin. Human Protein Atlas data show that GPR146 is also expressed in extrahepatic tissues. Cholesin is a secreted protein and is always present in the circulation. Does cholesin bind to GPR146 in these extrahepatic tissues and affect their physiological functions? (iii) Cholesin reduces the transcriptional activity of *SREBP2* in mouse liver, which is likely to reduce hepatic *LDLR* expression because SREBP2 upregulates *LDLR* expression at the transcriptional level. LDLR is primarily responsible for clearing circulating LDL-C. Will cholesin affect the clearance of LDL-C, thereby reducing its cholesterol-lowering effect? Answering these questions will help us further understand the physiological and pathological functions of cholesin and provide further evidence for its therapeutic potential.
